# Supplementary material for: Marine heatwaves shift ocean net primary productivity from the tropics toward the poles
Source: Nat Commun. 2026 Mar 31;17:4624. doi: 10.1038/s41467-026-71238-w (PMC13199387; doi:10.1038/s41467-026-71238-w)
Supplement: Supplementary file 1 — Supplementary Information [file 41467_2026_71238_MOESM1_ESM.pdf]

Supplementary Information

Marine Heatwaves Shift Ocean Net Primary Productivity from the Tropics to the Poles

Ce Bian[1], Zijie Zhao[2, 3, 4], Neil J. Holbrook[3, 4, 5], Peter G. Strutton[3, 4], Lixin Wu[6,7]

[1]Lamont Doherty Earth Observatory, Columbia University, New York, USA

[2]Department of Earth System Science, University of California, Irvine, CA, USA

[3]Institute for Marine and Antarctic Studies, University of Tasmania, Hobart, Australia

[4]Australian Research Council Centre of Excellence for Climate Extremes, University of Tasmania, Hobart, Australia

[5]Australian Research Council Centre of Excellence for the Weather of the 21st Century, University of Tasmania, Hobart, Australia

[6]Frontiers Science Center for Deep Ocean Multispheres and Earth System and Key Laboratory of Physical Oceanography, Ocean University of China, Qingdao, China.

[7]Laoshan Laboratory, Qingdao, China

Corresponding author: Ce Bian (cebian@ldeo.columbia.edu)

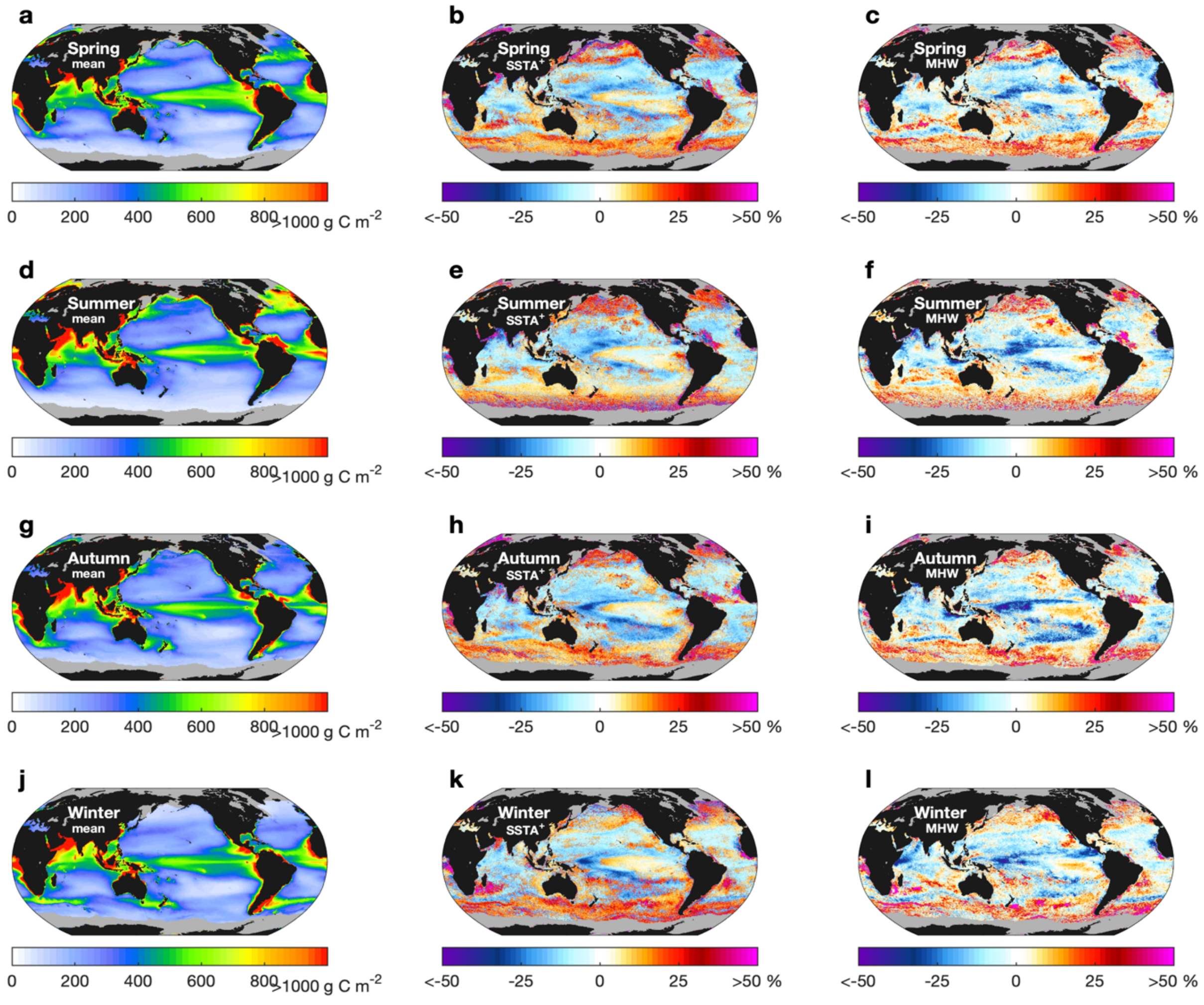

Supplementary Figure 1 | Seasonal characteristics of ocean net primary productivity (NPP) and related environmental factors during ($SSTA^{+}$) and marine heatwaves (MHWs). Same as Fig 1a-c, (a) is the mean pattern of NPP in spring, (b) and (c) are the NPP anomaly in spring during $SSTA^{+}$ and MHWs. Same as a-c, (d-f) show the corresponding results in summer, (g-i) in autumn, and (j-l) in winter.

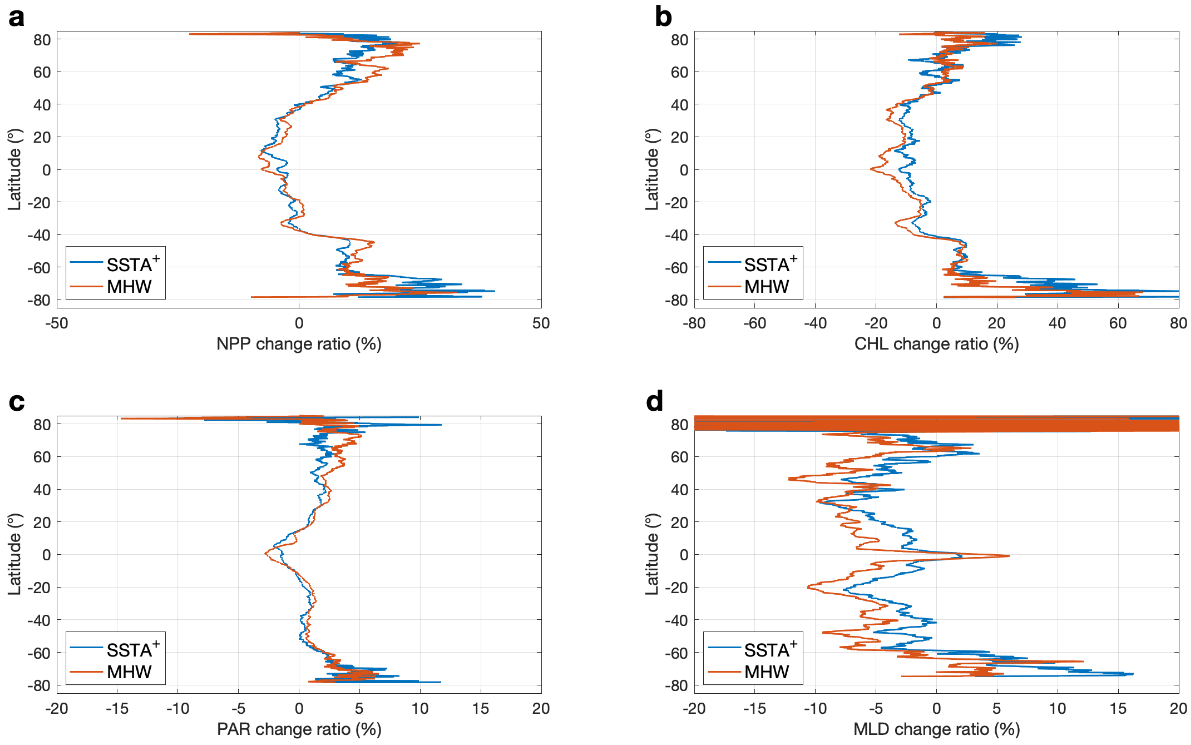

Supplementary Figure 2 | Latitudinal distributions of zonal-mean changes in ocean biogeochemical and physical variables. (a) Zonal-mean change ratios of NPP, defined as anomalies relative to the baseline mean state. (b–d) As in (a), but for chlorophyll-a (CHL), photosynthetically active radiation (PAR), and mixed-layer depth (MLD), respectively. Blue lines denote results during $SSTA^{+}$ and red lines denote results during MHWs.

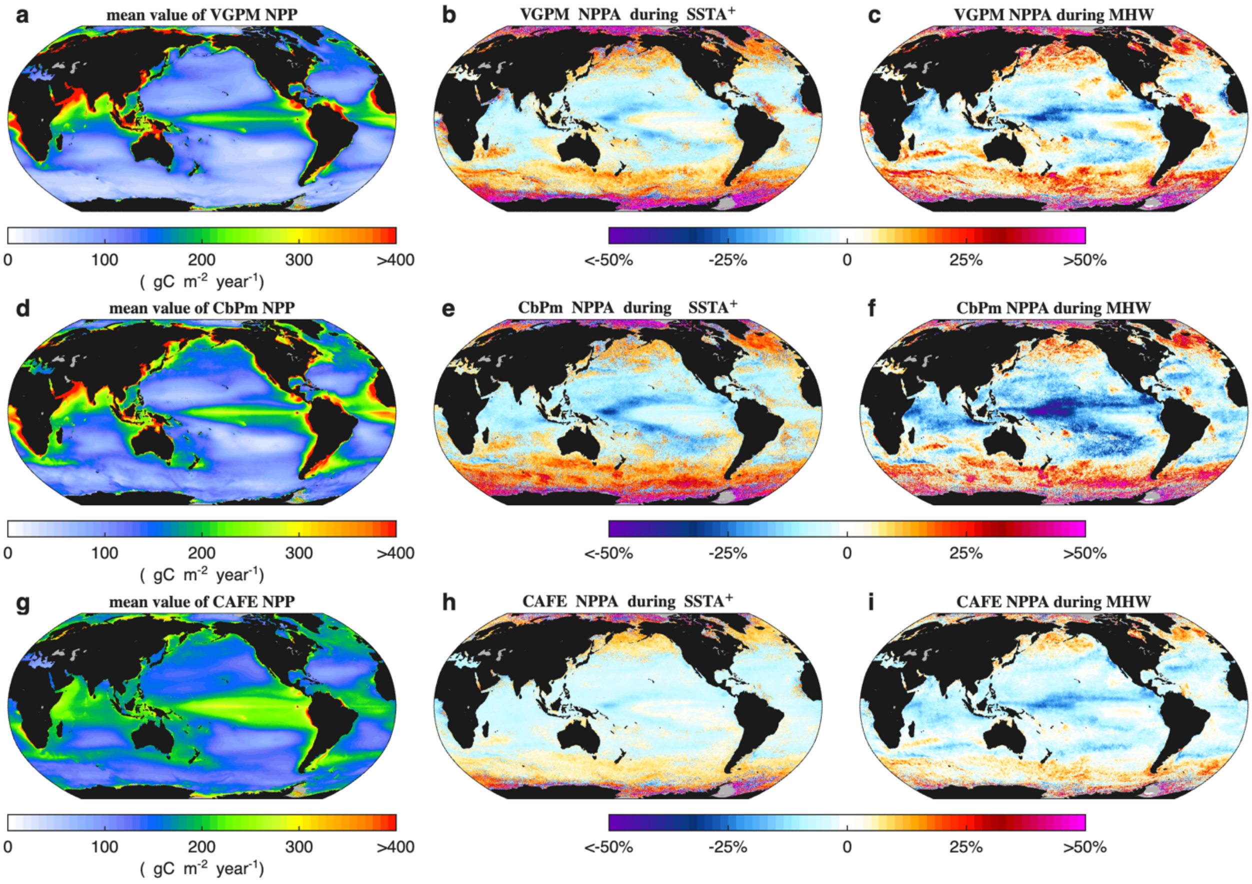

Supplementary Figure 3 | NPP products and their response under different warm condition. (a-c) is the same as Fig. 1a-c, (a) represents the mean spatial pattern of mean VGPM NPP during 1988–2018, (b) and (c) show the ratio of NPP changes compared with their local mean value during $SSTA^{+}$ periods and MHWs, respectively. Same as (a-c), but (d-f) applied to NPP products applied the updated Carbon-based Production Model (CbPM)[1,2]. (g-l) are applied to the NPP product based on method of Carbon, Antecedent conditions, Food web, and Ecosystem functioning model (CAFÉ)[3].

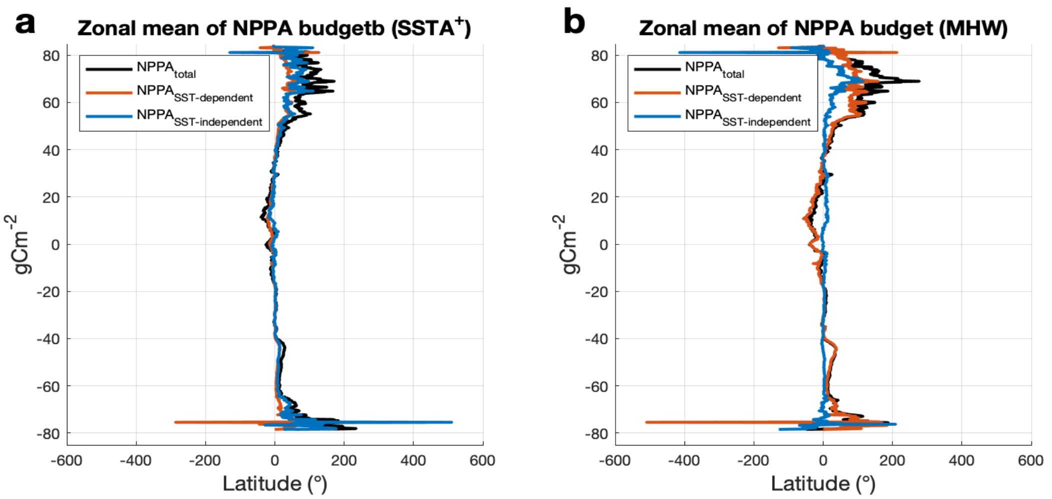

Supplementary Figure 4 | Latitude distribution of NPPA budget. This figure presents the zonal mean NPPA budget based on Fig 2. (a) The black line represents the total NPPA zonal mean value during the general $SSTA^{+}$ period, with contributions from SST-dependent terms (red) and SST-independent terms (blue). (b) Same as (a) but for MHW periods.

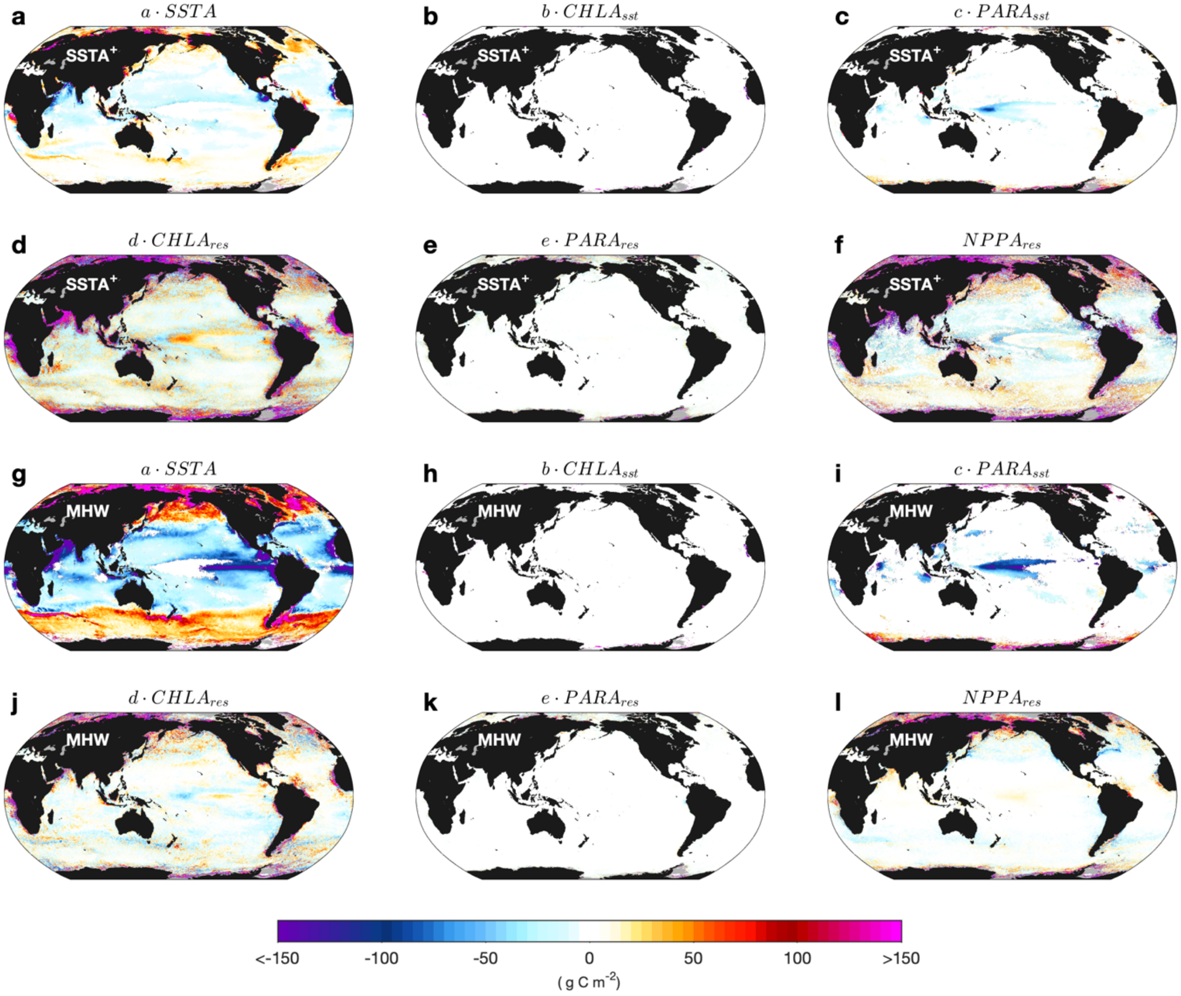

Supplementary Figure 5 | Contributions to NPPA according to terms in Eq. (2). (a-f) are results during $SSTA^{+}$ period, including contributions from linear SSTA (a), chlorophyll anomaly linearly related to SSTA (b), PAR anomaly linearly related to SSTA (c), SST-unrelated chlorophyll anomaly (d), SST-unrelated PAR anomaly (e), and unexplained part of NPPA (f) during general $SSTA^{+}$ periods. (g-k) same as (a-f) but during MHWs.

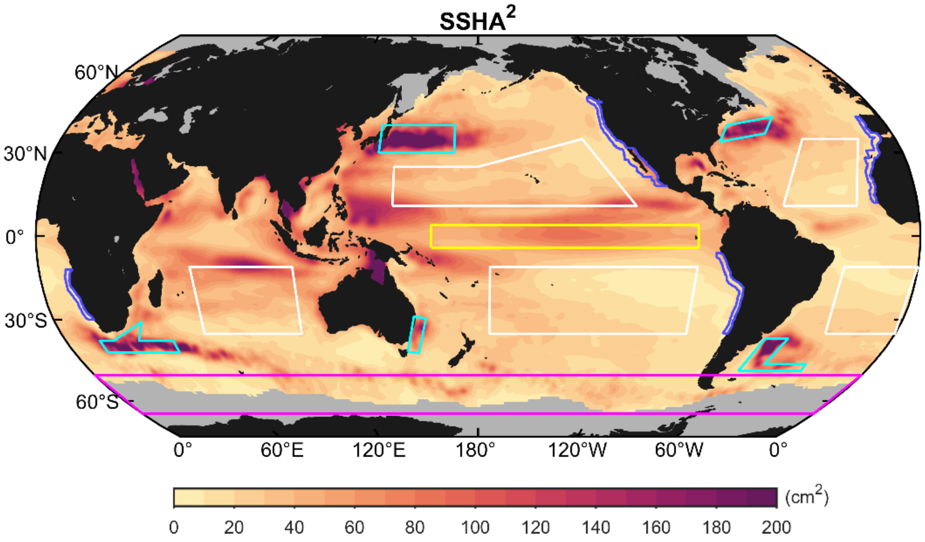

Supplementary Figure 6 | Domains of different regions. The western boundary currents and their extensions (encompassed by light blue line), the Southern Ocean (pink lines), the central-to-eastern equatorial Pacific (yellow lines), the eastern boundary upwelling systems (deep blue lines), and the subtropical gyre interior (white lines). Shading represents the variance of mesoscale sea surface height anomaly during 1993-2021 derived from the satellite altimeters. This figure is dapted from Bian et al. Nature Communications (2023), CC BY 4.0.

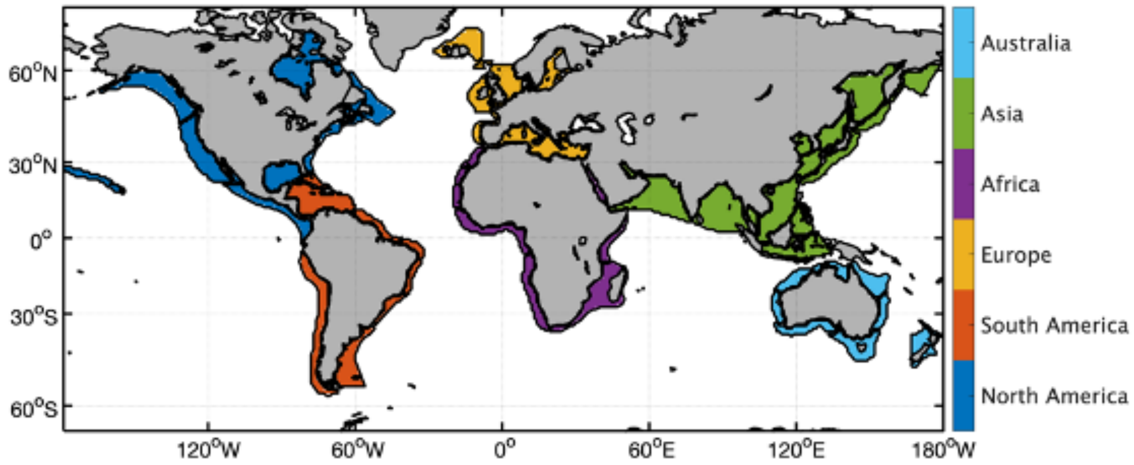

Supplementary Figure 7 | Classification of Large Marine Ecosystems (LMEs) by continent, including North America (blue), South America (orange), Europe (yellow), Africa (purple), Asia (green), and Australia (light blue). A total of 54 LMEs is categorized in this figure. The names of all 66 LMEs and their specific classifications are provided in Table S2.

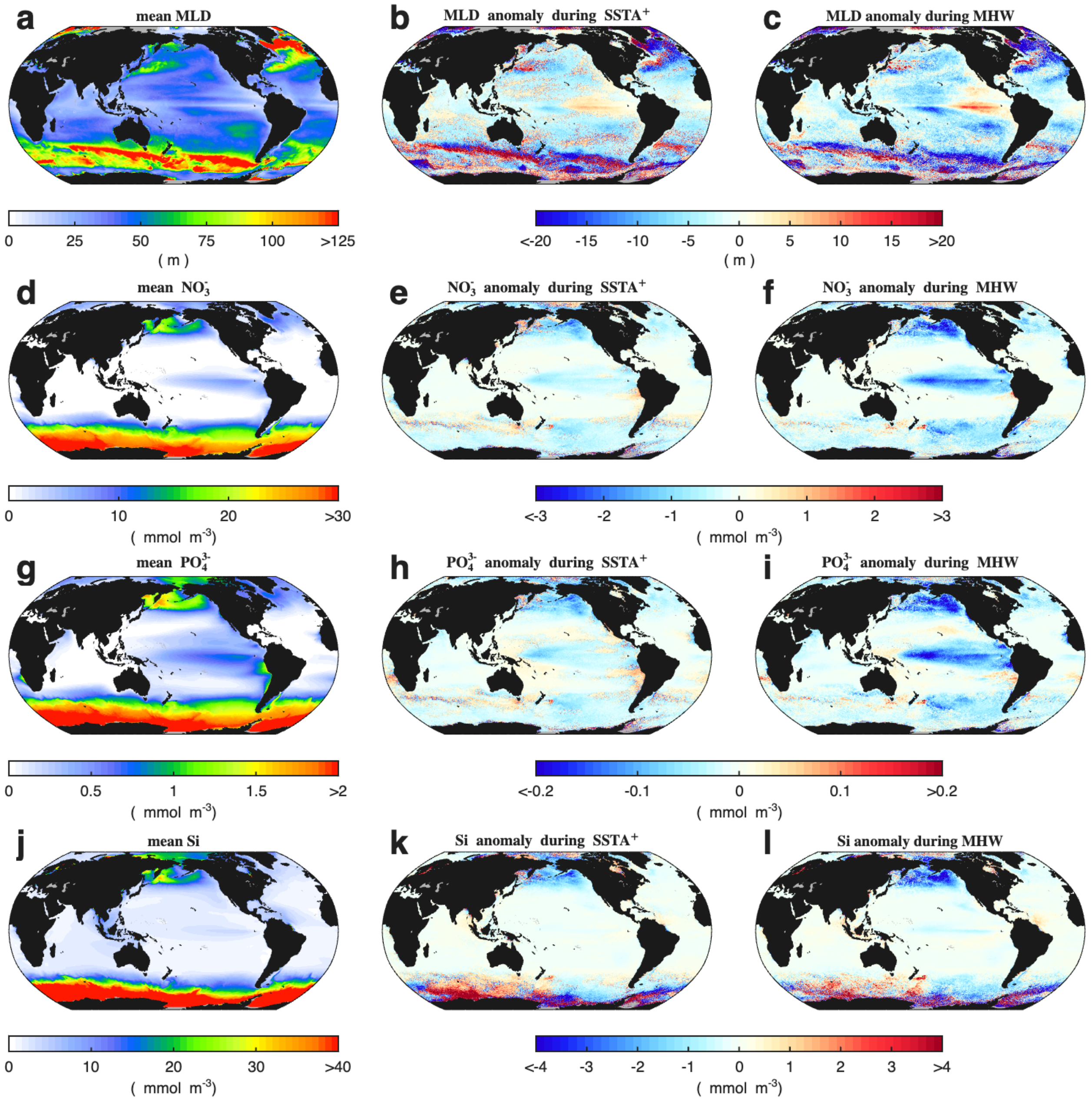

Supplementary Figure 8 | Spatial pattern of MLD and Nutrients during $SSTA^{+}$ and MHWs. (a) spatial pattern of mean MLD during 1988–2018. (b) and (c) are MLD changes during $SSTA^{+}$ periods and MHWs, respectively. Same as (a-c), (d-f) are mean nitrate concentrations (NO_3^{-}) and its corresponding change during $SSTA^{+}$ and MHWs period. (g-i) represent mean phosphate concentrations (PO_4^{3-}) and its anomaly during $SSTA^{+}$ and MHW periods. (j-l) represent mean silicate concentrations (Si) and its anomaly during $SSTA^{+}$ and MHW periods.

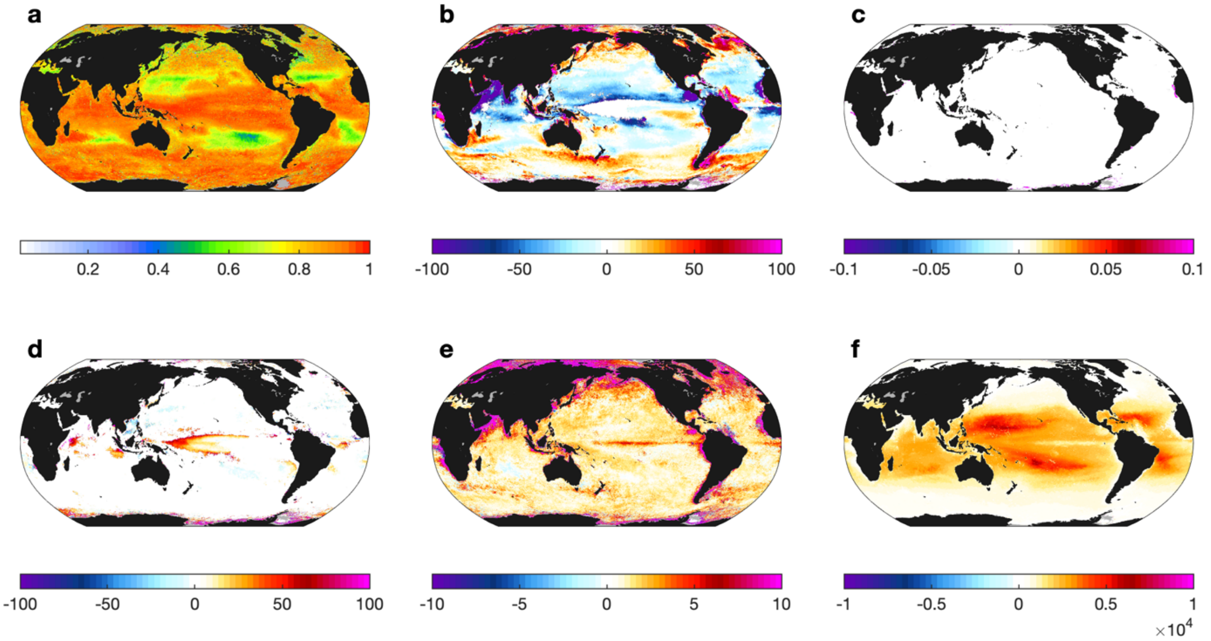

Supplementary Figure 9 | Evaluation of the NPPA Mathematical Model. (a) The R-squared value of the regression model (Eq. 2), where a value close to 1 indicates a high model fit, meaning the model explains nearly all the variability in the dependent variable and provides strong predictive capability. (b–f) The regression coefficients of the individual parameters (*a, b, c, d, and e*) in Eq. (2).

Supplementary Table 1 | Global LMEs and Their Continental Classification.

A comprehensive list of 66 Large Marine Ecosystems (LMEs) globally, categorized by continent following the classification used by Guo et al. Of these, 45 LMEs are continentally defined, consistent with Guo et al.'s framework.

LME Groups	Name List of LMEs	LME Groups	Name List of LMEs
North America	East Bering Sea,	Asia	Arabian Sea,
	Gulf of Alaska,		Bay of Bengal,
	California Current,		Gulf of Thailand,
	Gulf of California,		South China Sea,
	Gulf of Mexico,		Sulu-Celebes Sea,
	Southeast U.S. Continental Shelf,		Indonesian Sea,
	Northeast U.S. Continental Shelf,		East China Sea,
	Scotian Shelf,		Yellow Sea,
	Labrador-Newfoundland,		Kuroshio Current,
	Insular Pacific-Hawaiian,		Sea of Japan,
	Pacific Central American Coastal,		Oyashio Current,
	Hudson Bay Complex,		Sea of Okhotsk,
	Aleutian Islands		West Bering Sea
South America	Caribbean Sea,	Africa	Canary Current,
	Humboldt Current,		Guinea Current,
	Patagonian Shelf,		Benguela Current,
	South Brazil Shelf,		Agulhas Current,
	East Brazil Shelf,		Somali Coastal Current,

	North Brazil Shelf		Red Sea
Europe	North Sea,	Others	Central Arctic,
	Baltic Sea,		Canadian High Arctic - North Greenland,
	Celtic-Biscay Shelf,		Canadian Eastern Arctic - West Greenland,
	Iberian Coastal,		Greenland Sea,
	Mediterranean Sea,		Barents Sea,
	Iceland Shelf and Sea,		Norwegian Sea,
	Faroe Plateau,		Northern Bering - Chukchi Seas,
	Black Sea		Beaufort Sea,
Australia	North Australian Shelf,		East Siberian Sea,
	Northeast Australian Shelf,		Laptev Sea,
	East Central Australian Shelf,		Kara Sea,
	Southeast Australian Shelf,		Antarctica
	Southwest Australian Shelf,		
	West Central Australian Shelf,		
	Northwest Australian Shelf,		
	New Zealand Shelf		

Supplementary Table 2 | Ratio of area that dominated by SST-dependent processes.

The ratio (0-100%) is the area that leading by SST-dependent processes divide the total area at that type of region. Region definition is in Fig. S6.

**Region**	$\boldsymbol{SSTA^{+}}$	**MHW**
Global ocean	16%	79%
Global ice region	1%	87%
Western Boundary Currents and their Extensions	5%	94%
Eastern Boundary Upwelling Systems	1%	97%
Central-to-Eastern Tropical Pacific	21%	70%
The Southern Ocean	4%	69%
Subtropical Gyre Interiors	16%	77%

References

1. Westberry, T., Behrenfeld, M. J., Siegel, D. A. & Boss, E. Carbon-based primary productivity modeling with vertically resolved photoacclimation. *Global Biogeochemical Cycles* **22**, 2007GB003078 (2008).
2. Behrenfeld, M. J., Boss, E., Siegel, D. A. & Shea, D. M. Carbon-based ocean productivity and phytoplankton physiology from space. *Global Biogeochemical Cycles* **19**, 2004GB002299 (2005).
3. Silsbe, G. M., Behrenfeld, M. J., Halsey, K. H., Milligan, A. J. & Westberry, T. K. The CAFE model: A net production model for global ocean phytoplankton. *Global Biogeochemical Cycles* **30**, 1756–1777 (2016).
4. Bian, C. *et al.* Oceanic mesoscale eddies as crucial drivers of global marine heatwaves. *Nat Commun* **14**, 2970 (2023).
